# A COVID-19 Patient Presenting With Paroxysmal Atrial Fibrillation

**DOI:** 10.7759/cureus.17569

**Published:** 2021-08-30

**Authors:** Ioannis Nouskas, Vasiliki Holeva, Eleni Parlapani, Vasiliki Aliki Nikopoulou, Ioannis Diakogiannis

**Affiliations:** 1 Department of Cardiology, Affidea Medical Diagnostic Center, Thessaloniki, GRC; 2 Department of Psychology, Papageorgiou General Hospital, Thessaloniki, GRC; 3 1st Department of Psychiatry, Aristotle University of Thessaloniki, Thessaloniki, GRC

**Keywords:** covid-19, atrial fibrillation, inflammation, anxiety disorder, intensive care and invasive monitoring

## Abstract

The cardiovascular system is influenced in the course of coronavirus disease 2019 (COVID-19); paroxysmal atrial fibrillation (PAF) is not uncommon in hospitalized patients with COVID-19. This is a report of an atypical presentation of a 78-year-old patient who was diagnosed with COVID-19 infection. The patient, in the acute setting, was diagnosed with rapidly deteriorating cardiac failure associated with PAF, respiratory distress, and deteriorating vitals, and was eventually intubated. The mechanisms and preexisting substrates of atrial fibrillation in COVID-19 patients are discussed. A connection between arrhythmia and COVID-19, on the basis of a generalized inflammatory state, is suggested. This particular case adds to the understanding that the occurrence of PAF in COVID-19 patients is consistent with the mechanism of worse outcomes due to systemic inflammation.

## Introduction

Coronavirus disease 2019 (COVID-19) has quickly become a worldwide issue, primarily due to its contagious nature. It has been postulated that there are various phases of COVID-19: the first characterized by an incubation period associated with mild upper respiratory tract symptoms and fever, a second phase when the virus replicates and affects pulmonary function, and the third phase of systemic hyperinflammation with systemic effects on the liver, heart, kidney, intestines, and nervous system [1]. We present an atypical case of paroxysmal atrial fibrillation (PAF) and pulmonary edema in a 78-year-old obese Caucasian male with a documented past medical history of hypertension, coronary disease, and an anxiety disorder during the acute phase of severe acute respiratory syndrome coronavirus 2 (SARS-CoV-2) infection. The patient rapidly deteriorated and was transferred to an ICU where his hypoxia worsened prompting intubation. After seven days in the ICU, his clinical condition progressively improved and ventilatory support was gradually weaned. He was extubated on day 11 of admission. Normal sinus rhythm was restored two days prior to extubation. A connection is suggested between the arrhythmia and COVID-19 on the basis of a general inflammatory state [2]. As the COVID-19 pandemic progresses, more will be learned about the long-term cardiac sequelae of this virus. A prolonged, decreased functional status - compared to healthy individuals - cannot be excluded based on current data.

## Case presentation

**Figure 1:**
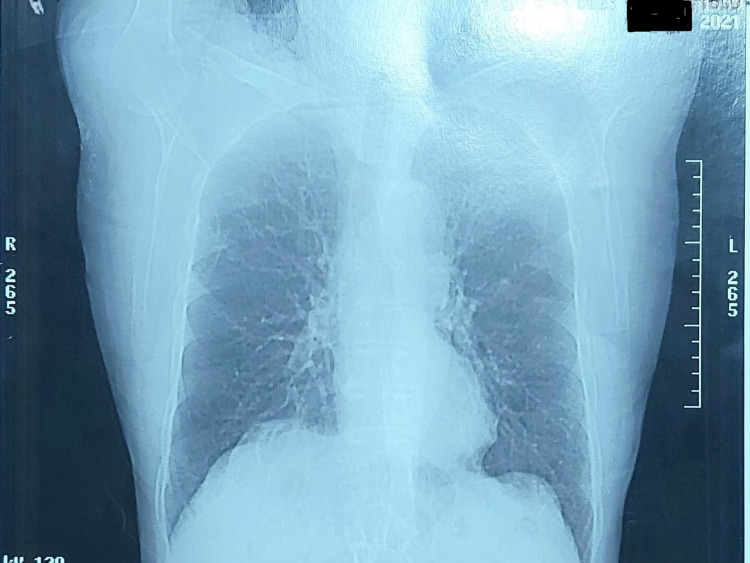
Chest X-ray revealed diffuse pulmonary edema, bilateral interstitial infiltrates and hazy opacities

A 78-year-old Caucasian male patient with a documented past medical history of arterial hypertension, coronary artery disease, a body mass index of 37, and an anxiety disorder, presented to our outpatient facility for diagnostic evaluation and appropriate management of his deteriorating respiratory distress. The patient presented with altered mental status in a state of a panic attack with palpitations, nausea, numbness throughout the body, tachypnea, heavy breathing, dry cough, and chest discomfort. The patient’s dyspnea was deteriorating. Vitals demonstrated a blood pressure of 137/71 mmHg, oxygen saturation of 83%, temperature of 39.7^o^C, heart rate of ~115 bpm, and a respiratory rate of ~33. The patient did not clinically require oxygen supplementation at the first phase of his evaluation during his initial work-up. Oxygen was subsequently rapidly supplemented and the patient was eventually intubated upon transfer to the ICU. Physical exam was notable for tachycardia with an irregular rhythm, a pulse deficit, and bibasilar crackles with no jugular venous distention or lower extremity edema. This was, historically, his very first episode of PAF. Chest X-ray revealed diffuse pulmonary edema, bilateral interstitial infiltrates, and hazy opacities (Figure 1).

**Figure 2:**
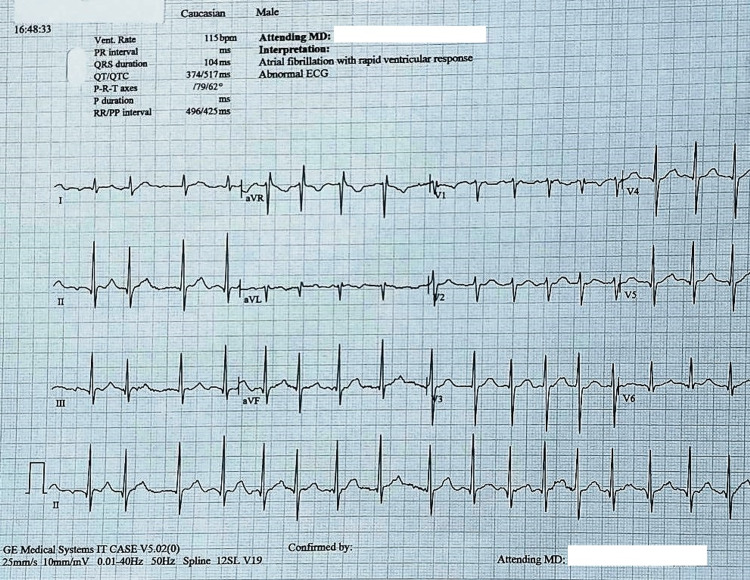
The initial electrocardiogram showing atrial fibrillation with a rapid ventricular response and repolarization abnormalities

On initial electrocardiogram, the patient was found to be in atrial fibrillation with rapid ventricular response and repolarization abnormalities (Figure 2).

**Figure 3:**
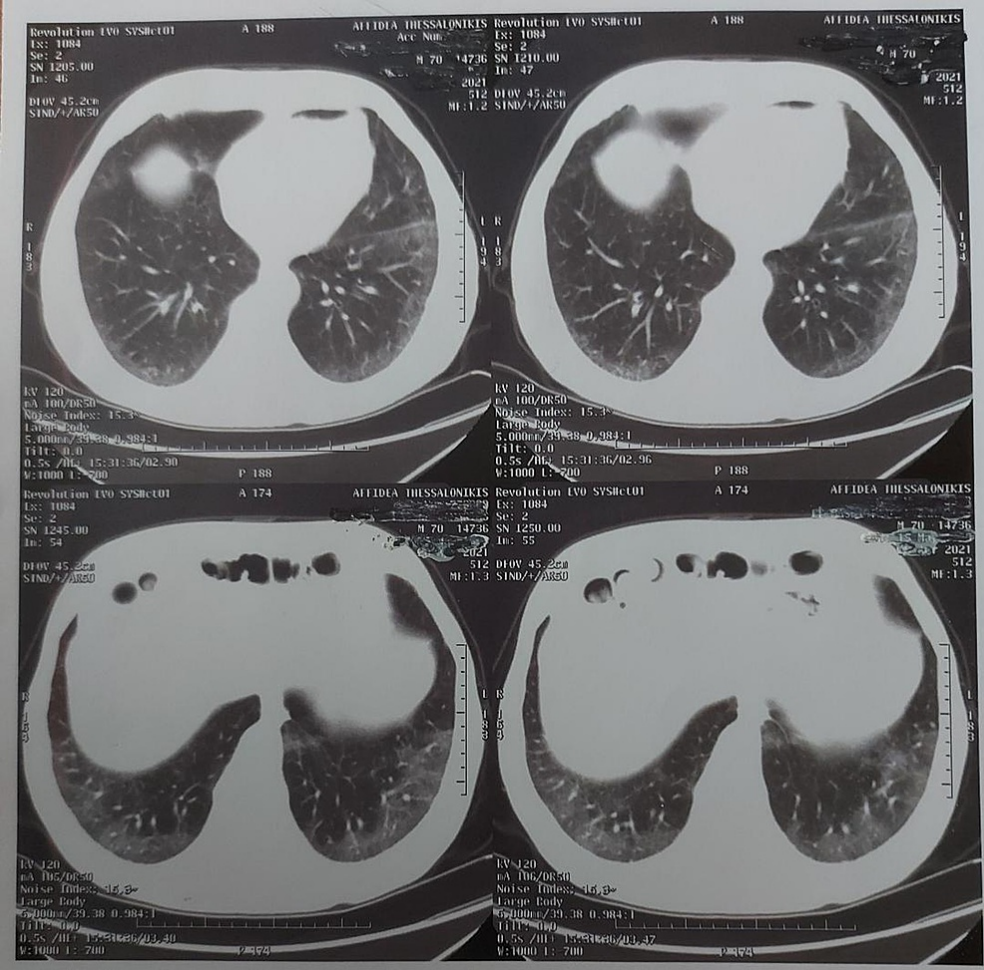
The CT scan of the chest demonstrating multiple areas of ground-glass opacities located predominantly peripherally and posteriorly

CT scan of the chest without IV contrast demonstrated multiple areas of ground-glass opacities located predominantly peripherally and posteriorly (Figure 3).

Labs revealed an anion gap of 23 mmol/L and a pro-B-natriuretic peptide level of 1863 pg/mL. Initial venous blood gas (VBG) demonstrated a pH of 7.51 with hypercapnia (pCO2 47 mmHg). Interpretation of acid-base disorders is based on arterial blood gas (ABG) rather than venous, but in critically ill adult patients, VBG may also be used to detect and diagnose acid-base disturbances with reasonable diagnostic accuracy compared to ABG [3]. The anion gap test was ordered because the patient had symptoms of high blood acid levels, i.e. shortness of breath, an abnormal heartbeat, and confusion. The anion gap measured was abnormal, indicating serious cardiovascular deregulation. Sodium was measured at 144 mEq/L and potassium at 4.4 mEq/L. In our patient, metabolic alkalosis apparently contributed to hypercapnic respiratory distress, acutely exacerbating his cardiac failure. Troponins were negative.

**Figure 4:**
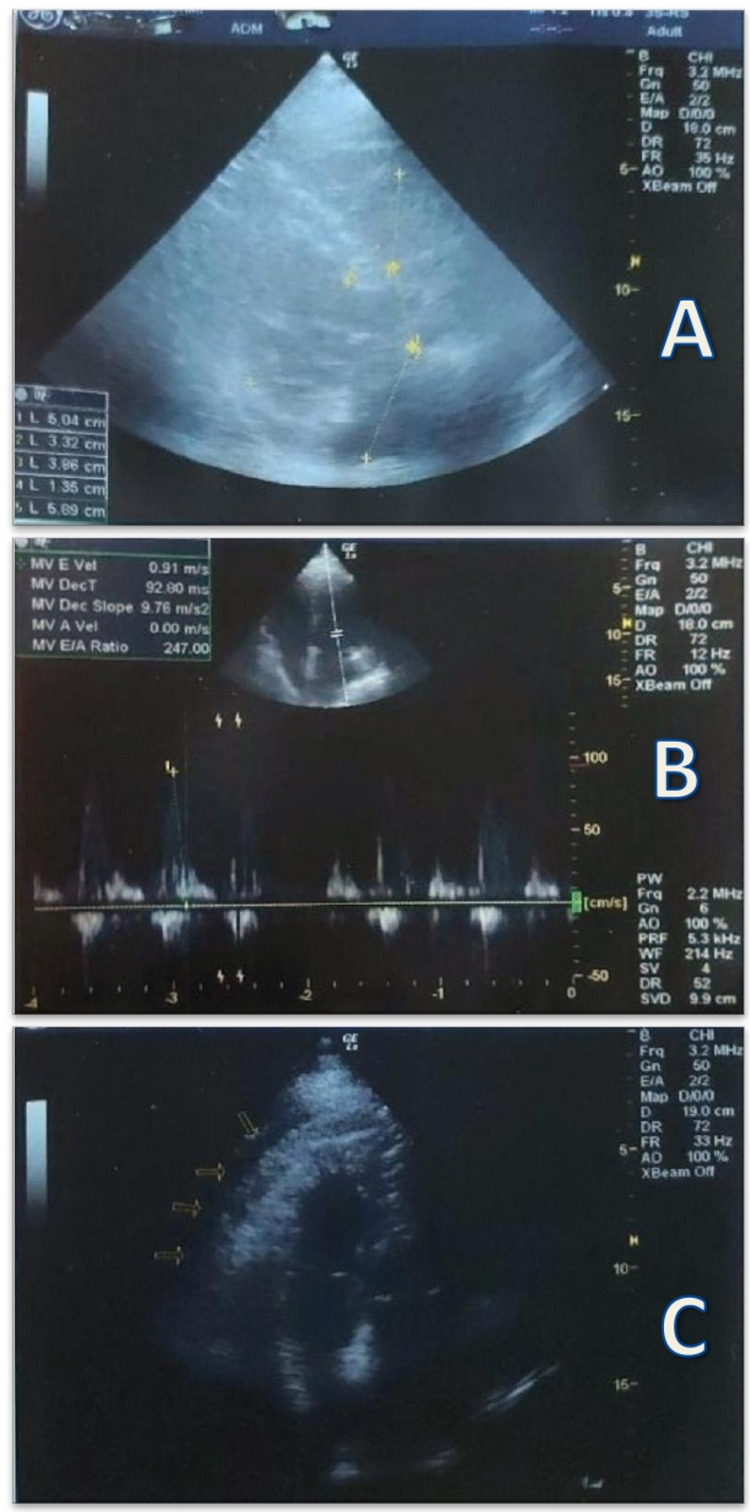
Echocardiography revealed an LV-ejection fraction of 50%-55% with grade III left ventricular diastolic dysfunction, biatrial and biventricular dilatation, a mild pericardial effusion, and a moderate septal hypertrophy (A) Parasternal long-axis imaging depicting biventricular and left atrial dilatation and moderate septal hypertrophy. (B) Pulsed wave Doppler study of transmitral inflow in atrial fibrillation, indicating early transmitral flow deceleration time (DT). E represents peak LV early diastolic filling velocity. Peak E velocity and DT vary depending on cardiac cycle length. Grade III left ventricular diastolic dysfunction. (C) Apical four-chamber view depicting a mild pericardial effusion.

Echocardiography revealed a borderline abnormal LV-ejection fraction of 50%-55% with grade III (restrictive) left ventricular diastolic dysfunction, biatrial and biventricular dilatation, a mild pericardial effusion, and moderate septal hypertrophy (Figure 4). Echocardiography in the acute setting also confirmed a mildly dilated LV (left ventricular end-diastolic diameter (LVEDD) was 58.9 mm and left ventricular end-systolic diameter (LVESD) was 40.7 mm). Transmitral E wave deceleration time was measured at 92.8 msec, a value indicative of a restrictive LV-filling pattern due to decreased ventricle compliance and severely elevated filling pressures.

COVID-19 was a novel inflammatory substrate tied to paroxysmal arrhythmia. No other precipitating factor for the present acute heart failure episode could be identified. According to the patient’s referring physician, his chronic heart condition has been well compensated, the patient has not been noticing any problems, and his occasional symptoms were fairly easy to manage.

Based on the patient’s clinical and imaging data, real-time reverse transcriptase-polymerase chain reaction (RT-PCR) was performed from a throat swab specimen with a positive result for SARS-CoV-2. The positive COVID test was obtained prior to clinically linking the patient's PAF to a COVID-19 infection.

**Figure 5:**
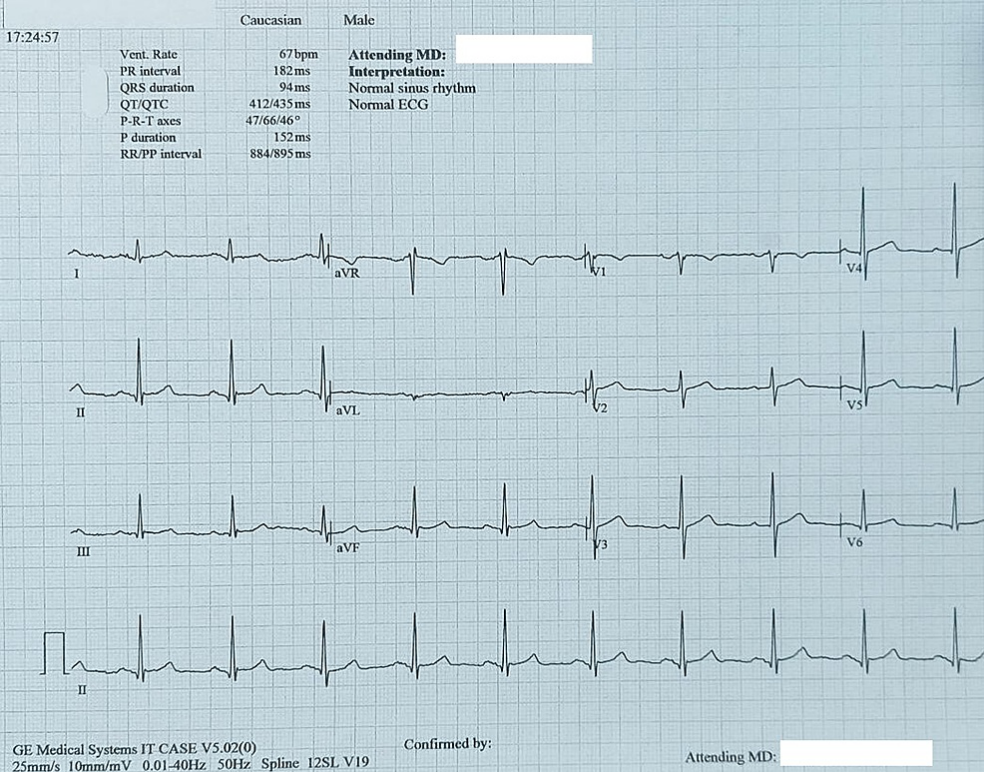
Restoration of normal sinus rhythm on the ECG prior to extubation

The patient rapidly deteriorated, was transferred to an ICU where he became hypoxic prompting intubation and vasopressor administration. The patient was treated with supportive care, dexamethasone, hydroxychloroquine, remdesivir, anticoagulation, and azithromycin. After seven days in the ICU, his clinical condition progressively improved and ventilatory support was gradually weaned. He was extubated on day eleven of admission. Normal sinus rhythm was restored two days prior to extubation, i.e. a total of nine days after initial presentation, including the period of ICU (Figure 5). Ibutilide IV infusion was administered in repeated doses, with no success.

## Discussion

Atrial fibrillation can be induced by a systemic inflammatory response and increased sympathetic tone [2]. PAF in COVID-19 may be tied to inflammation. Although COVID-19 is mostly characterized by respiratory symptoms, cardiovascular pathology and cardiac complications frequently arise in COVID-19 patients, increasing morbidity and mortality [4].

PAF is not uncommon in hospitalized patients with COVID-19 [5], suggesting a connection between the arrhythmia and COVID-19 on the basis of a general inflammatory state. It is not yet entirely clear whether SARS-CoV-2 actually has a direct myocardial effect causing arrhythmias. The incidence of PAF is increased in other severe respiratory syndromes as well, possibly contributing to increased mortality. PAF could be regarded as a significant, independent, negative biomarker that predicts mortality. The fact that it occurs in COVID-19 patients is consistent with the mechanism of worse outcomes due to the generalized inflammatory state.

COVID-19 patients with new-onset PAF are older, have increased inflammatory markers, including interleukin-6, more myocardial injury as assessed by troponin I, and higher mortality compared to patients without paroxysmal PAF [5]. Consequently, patients hospitalized with COVID-19 that develop PAF are considered to be at high risk. Their worse clinical outcome is probably not related to a direct myocardial effect of the virus, but should rather be attributed to the generalized inflammation [4]. Therefore, intensifying therapy with anti-inflammatory agents and antivirals should be considered in patients who present with COVID-19 and atrial tachyarrhythmia [6].

Mechanisms and pathophysiology of PAF in COVID-19 patients

The COVID-19 infection is an acute viral disease with an incubation period on average of five to seven days, in some cases up to 14 days. This relatively short time period is not, per se, sufficient to increase the risk of developing paroxysmal PAF by for instance causing myocardial fibrosis, which usually requires weeks to months to develop. While atrial structural remodeling is important in providing the PAF-inducing and -maintaining substrate, PAF onset and its paroxysms are often temporally related to acute COVID-19 infections.

Sinus tachycardia (fairly common in COVID-19 patients), myocardial injury, hypotension, bradycardia, and transient cardiomegaly have all been documented as complications of SARS-CoV-2 infection, particularly in patients with underlying cardiovascular pathology [7-9].

It is noteworthy that COVID-19 patients developing PAF are older and most of them have at least one preexisting risk factor, including arterial hypertension [10]. Older age and occurrence of congestive heart failure have also been associated with a greater likelihood of PAF during COVID-19 infection [11]. Therefore, COVID-19 patients with concomitant paroxysmal PAF may have a preexisting substrate for PAF and the acute COVID-19 infection possibly acts as a trigger for the initiation of PAF.

The pathophysiology of COVID-19-related PAF is not yet fully understood. Proposed mechanisms include viral endothelial damage, reduction in angiotensin-converting enzyme 2 receptor availability, CD147- and sialic acid-spike protein interaction, enhanced inflammatory signaling eventually culminating in an inflammatory cytokine storm, electrolytes and acid-base balance abnormalities, and a substantially increased adrenergic tone [12].

Although COVID-19 is known to increase inflammatory markers associated with atrial arrhythmias, the direct causal relationship of the inflammation to COVID-19 remains largely unknown, so a retrospective analysis comparing incidence, predictors, and outcomes of PAF in patients with COVID-19 is essential.

## Conclusions

The cardiovascular system is widely influenced in the setting of a severe COVID-19 infection. The suggested mechanism is indirect injury due to inflammation, endothelial stimulation, and microvascular thrombosis. Atrial fibrillation can be induced by a systemic inflammatory response and increased sympathetic tone. PAF in COVID-19 may be tied to inflammation. Cardiovascular pathology and cardiac complications frequently arise in COVID-19 patients, increasing morbidity and mortality. PAF is common in hospitalized patients with COVID-19, suggesting a connection between arrhythmia and COVID-19 on the basis of a general inflammatory state.
